# A systematic review with meta-analysis of the relation of aflatoxin B1 to growth impairment in infants/children

**DOI:** 10.1186/s12887-023-04275-9

**Published:** 2023-12-05

**Authors:** Behnam Ghorbani Nejad, Zahra Mostafaei, Ali Balouchi Rezaabad, Fatemeh Mehravar, Mahtab Zarei, Azadeh Dehghani, Mohammad Amin Raeisi Estabragh, Somayyeh Karami-Mohajeri, Hamzeh Alizadeh

**Affiliations:** 1 Department of Pharmacology and Toxicology, Faculty of Pharmacy, Kerman University of Medical Sciences, Kerman, Iran; 2 Department of Nutrition, Science and Research Branch, Islamic Azad University, Tehran, Iran; 3 Department of Pharmacognosy, School of Pharmacy, Kerman University of Medical Sciences, Kerman, Iran; 4 Department of Psychiatry and Community Health Nursing School of Nursing and Midwifery, Golestan University of Medical Sciences (GOUMS), Golestan, Iran; 5 Department of Cellular and Molecular Nutrition, School of Nutritional Sciences and Dietetics, Tehran University of Medical Sciences, Tehran, Iran; 6 Nutrition Research Center, Department of Community Nutrition, Faculty of Nutrition and Food Science, Tabriz University of Medical Sciences, Tabriz, Iran; 7 Pharmaceutics Research Center, Institute of Neuropharmacology, Kerman University of Medical Sciences, Kerman, Iran; 8 Genetics Research Center, Department of Genetics and Breeding, The University of Guilan, Rasht, Iran

**Keywords:** Infants, Children, Growth impairment, Aflatoxin

## Abstract

**Background:**

Aflatoxins are regarded as the most potent genotoxic and carcinogenic type of mycotoxins. This meta-analysis was performed to investigate a the relation of aflatoxin B1 (AFB1) to growth measurements of infants/children, including wasting, underweight, stunting, as well as weight-for-age (WAZ), height-for-age (HAZ), and weight-for-height (WHZ) z-scores.

**Methods:**

Electronic databases of PubMed, Web of Science, and Scopus were searched to identify related publications. Effect sizes for associations were pooled using the random effects analysis. Subgroup analysis by study design, method used to assess AFB1, and adjustment for covariateswas performed to detect possible sources of heterogeneity.

**Results:**

Pooled analysis of available data showed that AFB1 exposure was negatively associated growth z-scores, including WHZ (β = -0.02, 95%CI = -0.07 to 0.03), with WAZ (β = -0.18, 95%CI = -0.33 to -0.02), and HAZ (β = -0.17, 95%CI = -0.30 to -0.03) in infants/children. There was a remarkable heterogeneity among studies on WAZ and HAZ (*P* ≤ 0.001). In prospective cohort studies, AFB1 exposure was found to be significantly associated with the elevated risk of underweight (OR = 1.20, 95%CI = 1.03 to 1.40) and stunting (OR = 1.21, 95%CI = 1.11 to 1.33).

**Conclusions:**

This meta-analysis highlighted the importance of AFB1 exposure as a potential risk factor for growth impairment in infants/children.

**Supplementary Information:**

The online version contains supplementary material available at 10.1186/s12887-023-04275-9.

## Background

Produced by filamentous fungi, mycotoxins, are low-molecular-weight metabolites representing a public health concern worldwide. The global prevalence of mycotoxin contamination of food crops is estimated between 60 and 80% [1]. Aflatoxins (AFs) are regarded as the most potent genotoxic and carcinogenic type of mycotoxins mainly produced by *Aspergillus flavus and Aspergillus parasiticus.* These toxic metabolites could mostly flourish in food products such as rice, maize, groundnuts, legumes, and other grains, especially in tropical and subtropical regions, when crops are under stress (e.g. drought) or poor storage conditions (e.g. humidity) [2]. There are four main aflatoxin chemotypes (B_1_, B_2_, G_1,_ and G_2_), among which aflatoxin B_1_ (AFB_1_) is the most frequent and carcinogenic analog [3]. Chronic exposure to AFB_1_ could pose several adverse effects on the human body since upon its ingestion, AFB1 could convert to highly reactive metabolites i.e. exo-AFB1 8, 9-epoxide. If not detoxified, these compounds could bind to deoxyribonucleic acid (DNA) or protein molecules such as albumin, leading to immune suppression [4], hepatocellular carcinoma [5], and childhood growth impairment [6]. AFB1 can be measured using various analyses, such as enzyme-linked immunosorbent assay (ELISA), high-performance liquid chromatography (HPLC), thin layer chromatography (TLC), and isotope dilution mass spectrometry (IDMS) [6].

Child growth failure characterized by stunting (height-for-age Z-score (HAZ) < -2 standard deviations (SDs), wasting (weight-to-height Z-score (WHZ) < -2 SDs, underweight (weight for age Z-score (WAZ) < -2 SDs has still remained a serious problem in many low- and middle-income countries [7]. According to the World Health Organization (WHO), impaired child growth as a result of nutrient deficiencies, recurrent infections, and environmental toxins is responsible for about 45% of under-5 mortality worldwide. This could also exert detrimental effects on children's cognitive, metabolic, and physical development leading to serious health consequences later in life [8].

Accumulating evidence suggested long exposure to AFB1 could be a potential risk factor for poor growth among children, especially in countries that are more susceptible to AFs contaminations [9, 10]. However, the biological mechanisms through which AFB1 exposure may affect child growth are not fully understood. It has been hypothesized that exposure to aflatoxins may impair intestinal integrity, disturb liver metabolism, and inhibit the synthesis of proteins including insulin-like growth factor 1 (IGF-1), thus contributing to malabsorption, inflammation, and gut infection. Each of these effects could in turn impair child growth [11, 12]. Early exposure to Aflatoxin could occur during pre-natal and neonatal stages through transplacental transfer and breast milk. Once complementary feeding has started, the levels of AFB1 in children will significantly rise and reach as high as of those adults [13]. Therefore, early childhood is considered a critical stage to reduce aflatoxin exposure and prevent its long-lasting outcomes.

Existing literature regarding the causal association between AFB1 and child growth is limited and inconsistent. Previous epidemiological studies conducted on Gambia [14, 15], Nigeria [16], Kenya [17], Mexico [18], and Lebanon [19] reported AFB1 solely or in relation to other risk factors have negatively impacted child growth. However, other studies in Tanzania [20], Ethiopia [21], and Nepal [22] showed no relationship between AFB1 exposure and child growth impairment. Likewise, a study among Kenyan children suggested that improving household access to aflatoxin-free maize reduced aflatoxin biomarker concentration in serum, but it showed no significant effect on linear child growth [23]. The inconsistencies in the results of the previous studies may be resulted from the difference in study design, method used to assess AFB1, and the varieties in adjustment for covariates. With this regard, the present systematic review and meta-analysis aimed to summarize the findings on the association between AFB1 exposure and growth indicators of children in the overall and different subgroups.

## Materials and methods

This study was conducted based on the PRISMA guidelines [24] (Supplementary file 1).

### Search strategy

A comprehensive literature search was conducted in PubMed, Web of Science, and Scopus for relevant literature from the beginning to August 2023. A manual search was also performed to avoid missing an article. The search was conducted using the keywords related to AFB1 in children growth indicators such as height, weight, stunting, wasting, and underweight. The complete search strategies is reported in Supplementary file 2. No language limitation was considered for the search. All articles were entered into the Endnote software. After removing duplicates, their titles and abstracts were checked separately by two researchers and quality assessment was done and differences were resolved by group discussion with an involvement of a third researcher. Resolving disagreements among authors in terms of including studies in the meta-analysis was conducted by an open communication among the authors involved in the disagreement and by reviewing the inclusion/exclusion criteria. Then, articles related to the inclusion criteria were included. Then the full articles were downloaded and the information was entered in the extract table.*Inclusion criteria*The inclusion criteria were: 1) observational studies (cross-sectional, cohorts, case–control, nested case–control) reported AFB1 exposure (serum levels) in infants/children (≤ 12 years) in relation to growth measurements (stunting (HAZ < -2 standard deviations (SDs), wasting (WHZ < -2 SDs), underweight (WAZ < -2 SDs), HAZ, WAZ, and WHZ); 2) studies reported odds ratios (OR), hazard ratios (HR), relative risks (RR), or the standardized regression coefficient (β) and their 95% confidence intervals (CI) or raw data were available to calculate them. No limitation was considered for analysis methods used to measure AFBI in serum. Clinical and interventional studies on animals, review articles, methodology studies, animal and non-human studies were excluded. We developed a form based on the inclusion/exclusion criteria and evaluated the studies for their eligibility.

### Data extraction

Data extraction was accomplished independently on all studies by two authors. Disagreement were solved by discussion. The author’s name, publication year, study design, sample size, region, outcomes, mean age of participants, risk estimates for binary outcomes (stunting, wasting, underweight), standardized regression coefficient (β) and its 95%CI for continuous outcomes (growth z-scores), method used for AFB1exposure measurement, the level of adjustment for covariates and statistical models applied for data analyzes were extracted.

### Quality assessment

We used the Newcastle–Ottawa scale (NOS) to check the methodological quality of the studies [25]. A modified version of NOS was used for cross-sectional studies as reported previously [26]. NOS included 3 categories and 8 items that range from 0 to 9 stars. The three categories include selection, comparison, and result, which contain 4 stars, 2 stars, and 3 stars, respectively. High quality is a score greater than or equal to 6, medium quality is a score of 3 to 5, and low quality is a score of less than 3 [27].

### Statistical analysis

The relationship between infants/children exposure to AFB1 and continuous outcomes (growth z-scores) was evaluated by determining the standardized regression coefficient (β) and 95%CI. The relation between exposure to AFB1 and categorical outcomes (stunting, wasting, and underweight) was computed by pooling ORs and corresponding 95%CIs. I2 statistic was used to assess statistical heterogeneity across included studies [28–31]. The DerSimonian and Laird random effects model was used to perform all pooled results due to anticipated heterogeneity [32]. Subgroup analysis by study design (prospective cohort vs. non-cohort), method used to assess AFB1 (ELISA, HPLC, IDMS, and TLC), and adjustment for covariates (yes vs. no) was performed to detect possible sources of heterogeneity. For continuous outcomes, because quantitative variables have been reported with different statistical metrics (regression coefficient, correlation coefficient, or mean differences in the exposed and non-exposed groups), standard regression coefficients (β) and corresponding 95%CI (or standard error) were used to present a combination of the findings of different studies. The β and its 95%CI (or standard error) was obtained from the estimates whose calculation method is given in the Supplementary file 3. STATA software (version 14.0; Stata Corporation, College Station, TX, USA) was used for statistical calculations.

## Results

### Study characteristics

**Fig. 1:**
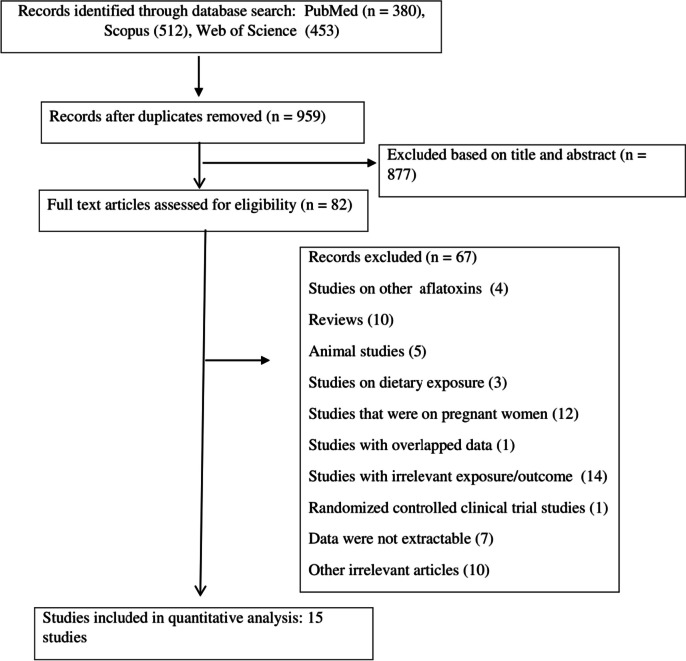
Flow chart for studies selection

The search strategy found 1345 publications and 386 papers were identified to be duplicates. A total of 877 articles excluded according to the titles and abstracts, yielding 82 potentially pertinent studies for the full-text evaluation. Ultimately, 15 publications [14–17, 21, 33–42], published between 2003 and 2023, with a total sample size of 5,511 subjects, were included in the meta-analysis. Figure 1 shows the flowchart of the screening process of studies based on the PRISMA guideline [43]. Studies were from 2 continents, 11 from Africa and 4 from Asia. Of these, 8 records were prospective cohort [14, 15, 21, 36, 38–40, 42] and 7 records were non-cohort [16, 17, 33–35, 37, 41] in design. The sample size of the included studies ranged between 46 and 1484 participants. AFB1 levels were measured using the ELISA in 5 studies [14, 15, 33, 35, 36], HPLC in 8 studies [16, 17, 21, 37–41], IDMS in one study [42], and TLC [34] in one study. The majority of the studies controlled the results for potential covariates, but, the results of 1 publication [34] was crude results without adjustment for confounders. Data for height, HAZ, WAZ, WHZ, stunting, underweight, and wasting were available in 4 studies, 12 studies, 8 studies, 6 studies, 6 studies, 3 studies, and 1 study, respectively. The methodological quality of the analyzed publications was medium to high, with scores ranging from 4 to 8 (Supplementary file 4). The characteristics of the included papers are presented in Table 1.

**Table 1:** Characteristics of studies

**Reference**	**Year**	**Study design**	**Location**	**N of participants**	**Age (rang or mean ± sd)**	**Exposure**	**Exposure assessment**	**Mean serum AFB1(mean** ± sd, or as mentioned)	**Outcomes**	**Statistical models**	**Adjustment**
Alamua et al.	2019	Cross-sectional	Zambia	311	6–24 months	AFB1-lysine (serum)	HPLC	0.21 ± 0.75 ng/mL	Stunting, underweight	Logistic regression	Sex, sickness, age, serum albumin
Andrews-Trevino et al.	2021	Prospective cohort	Nepal	1484	3 to 22 months	AFB1-lysine (serum)	HPLC	1.27 pg/mg albumin (95%CI: 1.18–1.36)	Stunting, height, WHZ, WAZ, HAZ	Logistic regression Linear regression	Age, season of measurement, and detectable AFB1 concentrations (yes/no)
Ashraf et al.	2022	Cross-sectional	Pakistan	238	1–11 years	AFB1-lysine (serum)	HPLC	11.27 pg/mg albumin (95%CI: 15.88–24.17)	Stunting, underweight, wasting	Logistic regression	Adjusted for potential covariates(not reported)
Mahfuz et al.	2020	Prospective cohort	Bangladesh	228	7–36 months	AFB1-lysine (serum)	HPLC	3.70 pg/mg albumin (range: 0.09 -126.54)	HAZ, Stunting	Logistic regression Linear regression	Sex, concentrations of myeloperoxidase in stool, low birth weight, maternal height, number of people sleep in one room, improved toilet, treatment of drinking water, asset categories
Andrews‐Trevino et al.	2021	Prospective cohort	Nepal	699	18–22 months	AFB1-lysine (serum)	HPLC	2.41 ± 7.88 pg/mg albumin	Stunting, underweight, height, WAZ, HAZ	Logistic regression Linear regression	Length, weight or anthropometric z‐scores at birth or head circumference at 3 months, child's minimum dietary diversity (yes/no) and mother's schooling
Shirima et al.	2015	Prospective cohort	Tanzania	166	6–14 months	AFB1-lysine (serum)	ELISA	4.7 pg/mg albumin (95%CI: 3.9—5.6)	Height, HAZ	Multivariable regression	Village, breastfeeding, maternal education, and family socioeconomic status and protein and energy intakes, sex, baseline age in month, and baseline length
Tessema et al.	2021	Prospective cohort	Ethiopia	102	6–35 months	AFB1-lysine (serum)	HPLC	NR	WHZ, HAZ	Linear regression	child’s sex, age, time of assessment (pre- or post-harvest), intervention arm, inflammation, household wealth tertile and incidence of diarrhea
Watson et al.	2018	Prospective cohort	Gambia	374	6–18 months	AFB1-lysine (serum)	ELISA	52.29 ± 5.61 pg/mg albumin	WHZ, WAZ, HAZ	Linear regression	Season of sampling, mother’s household quality, supplementation group, and age of introduction of non-breast milk foods
Turner et al.	2007	Prospective cohort	Gambia	138	Under 1 year	AFB1-lysine (serum)	ELISA	8.7 pg/mg albumin ( range: 5.0—30.2)	WAZ, HAZ	The generalized estimating equations multiple regression	Gender, age, placental weight, maternal weight, gestation duration and season
Gong et al.	2003	Cross-sectional	Benin and Togo	479	9 months to 5 years	AFB1-lysine (serum)	ELISA	32.8 pg/mg albumin (95% CI: 25.3—42.5)	WAZ, HAZ	Multivariable regression	Age, sex, socioeconomic status, agro ecological zone, and weaning status
McMillana et al.	2018	Case–control	Nigeria	58	6 – 48 months	AFB1-lysine (serum)	HPLC	2.6 pg/mg albumin (range: 0.2—59.2)	Stunting, WHZ, HAZ	Logistic regression Spearman's correlations	Age
Wangia-Dixon et al.	2020	Cross-sectional	Kenya	748	6–12 years	AFB1-lysine (serum)	HPLC	10.5 pg/mg albumin (95%CI: 9.4 -11.7)	WHZ, WAZ, HAZ	Linear regression	County, age of child, go to bed and feel hungry, age of mother when she had first child, marital status, living condition, and mother’s education
Shouman et al.	2011	Cross-sectional	Egypt	46	1 month to 4.5 years	AFB1 (serum)	TLC	51.61 ppm ( interquartile range: 30.565 -62.795)	WAZ, HAZ	Spearman’s correlation	Not adjusted
Castelino et al.	2015	Cross-sectional	Kenya	199	12.0 ± 3.0 years	AFB1-lysine (serum)	ELISA	110.5 pg/mg albumin (95%CI: 95.4 -127.9)	Height	Multivariable regression	Age, sex, school, liver disease state and infection status
Matchado et al.	2023	Prospective cohort	Malawi	241	Under 30 months	AFB1-lysine (serum)	IDMS	NR	WHZ, WAZ, HAZ	Linear regression	Maternal prepregnancy body mass index, HIV, weight and mid-upper-arm-circumference-for-age z-score gain rate from baseline to 36 weeks, housing quality, food security, child sex, age, malaria, hemoglobin

*AFB1* Aflatoxin B1, *WHZ* Weight-for-height z score, *WAZ* Weight-for-age z-score, *HAZ* Height-for-age z-scores, *HPLC* High-performance liquid chromatography, *ELISA* Enzyme-linked immunosorbent assay, *TLC* Thin layer chromatography, *IDMS* Isotope dilution mass spectrometry, *HIV* Human immunodeficiency virus, *NR* Not reported

### AFB1 exposure and growth measurements

**Fig. 2:**
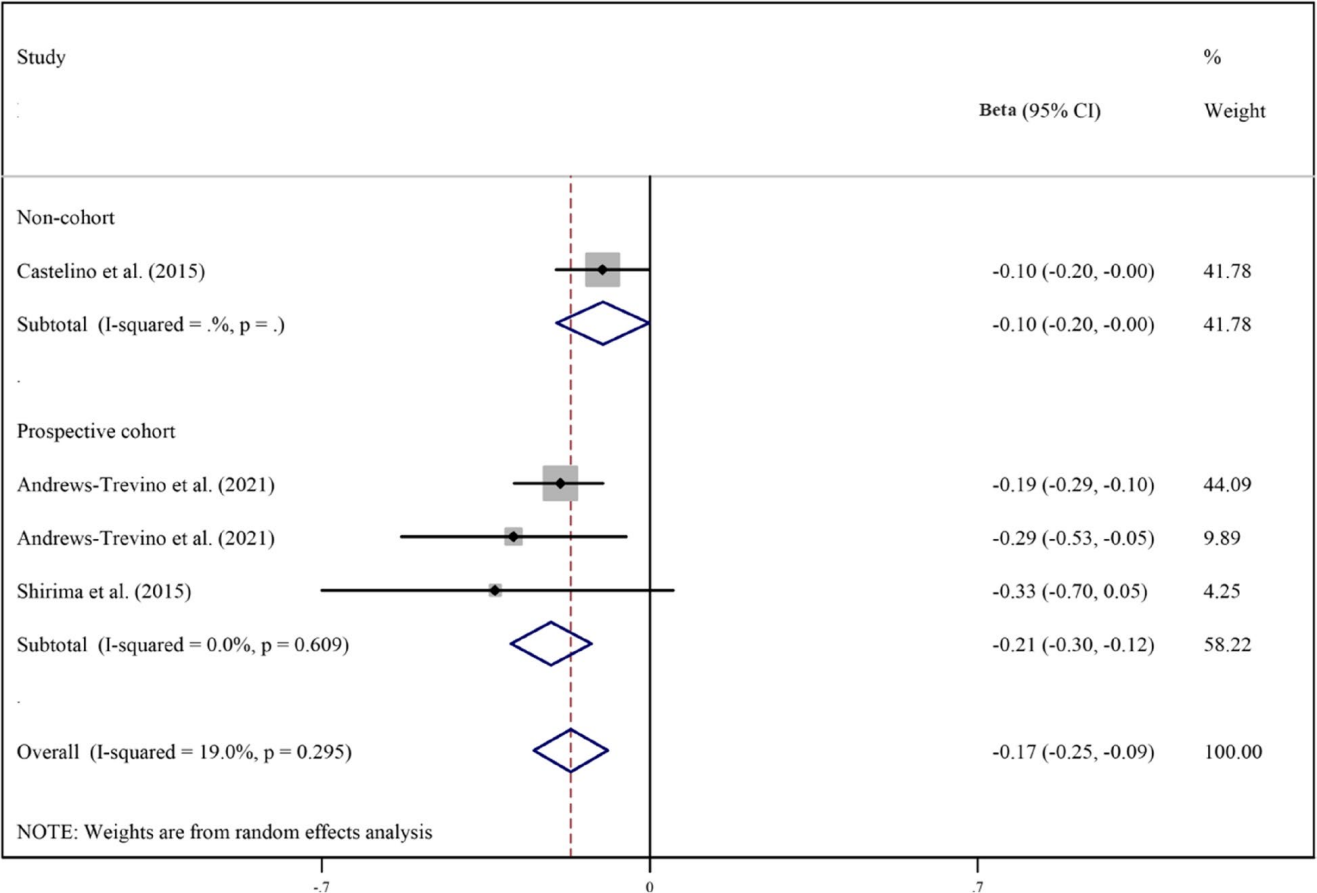
Meta‐analysis of the association between exposure to aflatoxin B1 and height of children stratified by study design (β coefficient and 95% confidence interval)

**Fig. 3:**
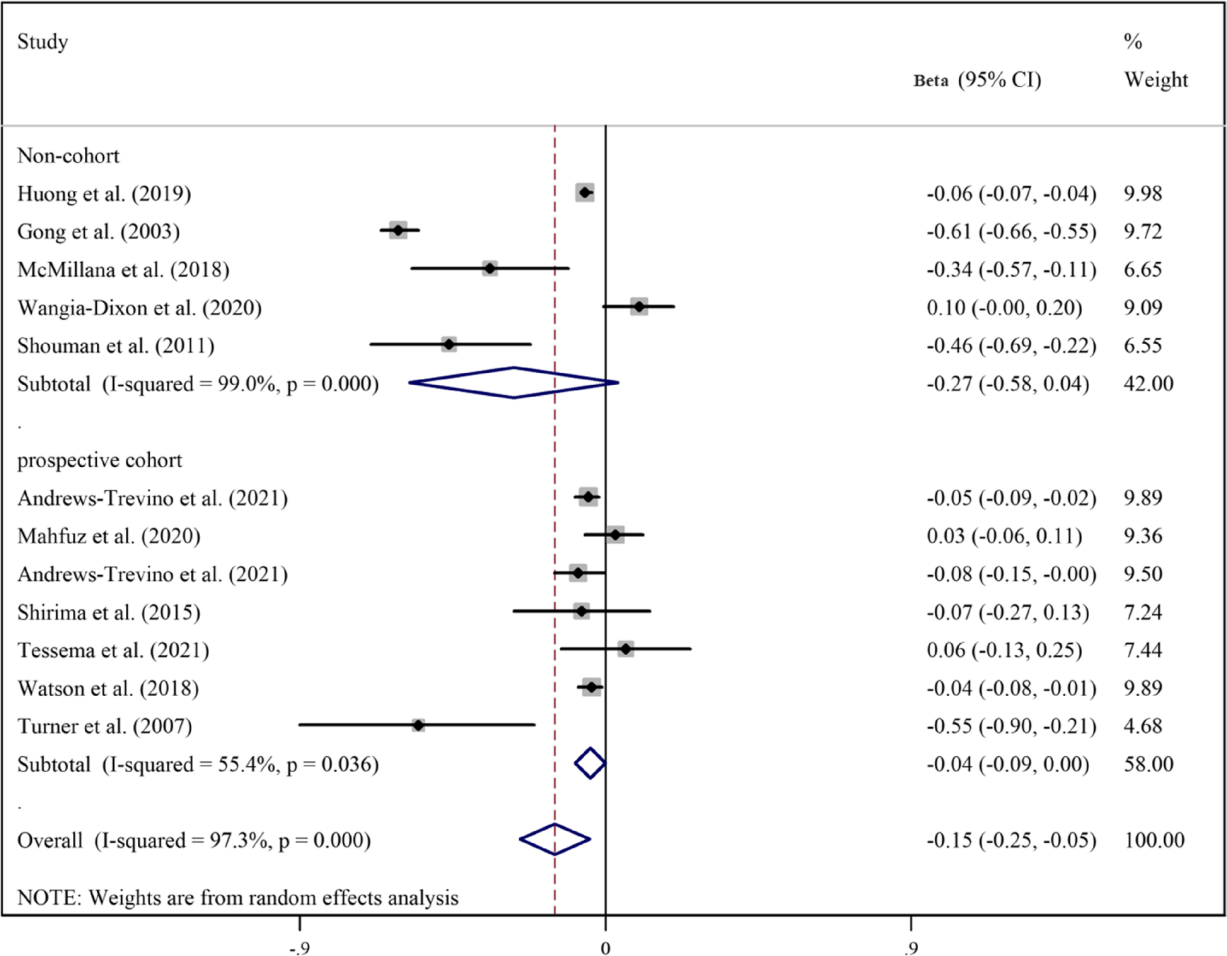
Meta‐analysis of the association between exposure to aflatoxin B1 and height-for-age z-score (HAZ) stratified by study design (β coefficient and 95% confidence interval)

**Fig. 4:**
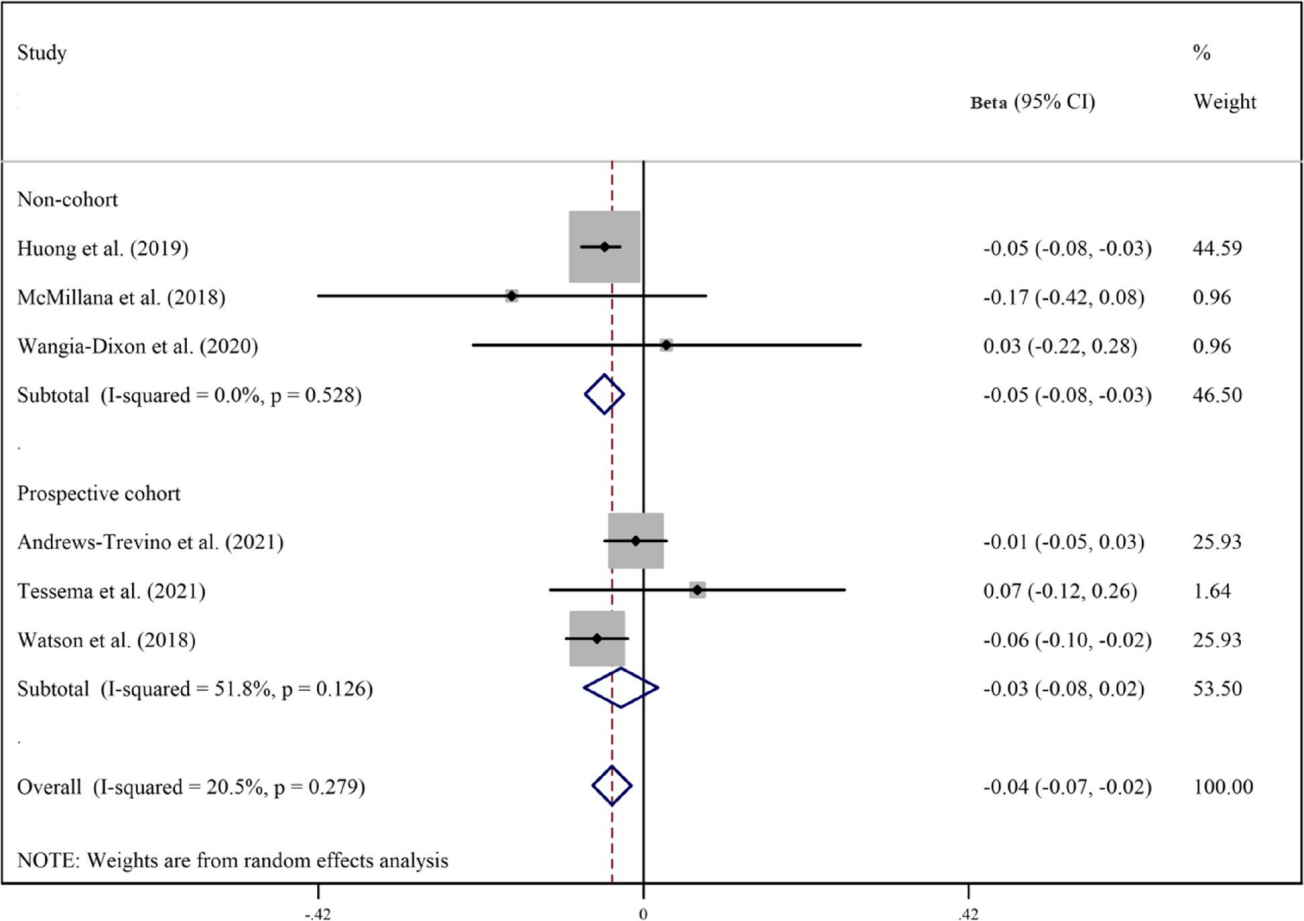
Meta‐analysis of the association between exposure to aflatoxin B1 and weight-for-height z score (WHZ) stratified by study design (β coefficient and 95% confidence interval)

**Fig. 5:**
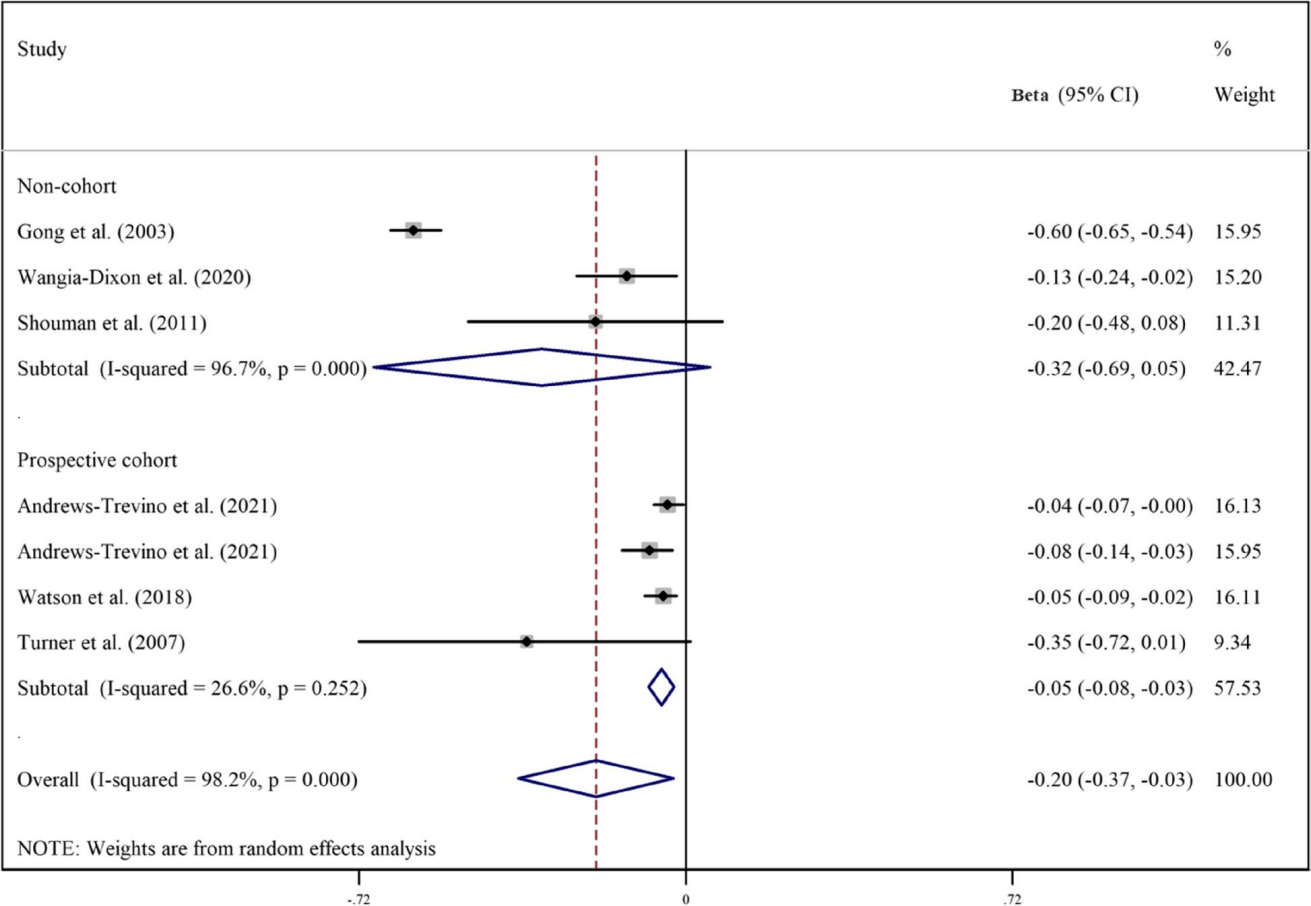
Meta‐analysis of the association between exposure to aflatoxin B1 and weight-for-age z score (WAZ) stratified by study design (β coefficient and 95% confidence interval)

Pooled analyzes of all available studies revealed a negative association between higher AFB1 exposure and height (β = -0.17, 95%CI = -0.25 to -0.09; Fig. 2), HAZ (β = -0.17, 95%CI = -0.30 to -0.03; Fig. 3), WAZ (β = -0.18, 95%CI = -0.33 to -0.02; Fig. 4), and WHZ (β = -0.02, 95%CI = -0.07 to 0.03; Fig. 5) in children, with a significant evidence of heterogeneity across studies on HAZ (I2 = 99.0%, *P* ≤ 0.001) and WAZ (I2 = 98.2%, *P* ≤ 0.001). The results of the subgroup analysis by study design (prospective cohort vs. non-cohort), method used to assess AFB1 (HPLC, ELISA, IDMS, and TLC), and adjustment for covariates (yes vs. no) is presented in Table 2. The inverse trend for the relation of AFB1 to height, HAZ, WHZ, and WAZ was also supported by the subgroups.

**Table 2:** Subgroup analysis for the association between aflatoxin B1 and growth indicators of children

					**Test of association**	**Test of heterogeneity**
**Continuous outcome**	**Subgroup**	**Studies**	**β**	**95%CI**	**I**^**2**^** (%)**	**P**
Height	Overall	4	-0.17	-0.25 to -0.09	19.0	0.29
	Prospective cohort	3	-0.21	-0.30 to -0.12	0.0	0.60
	Non-cohort	1	-0.10	-0.20 to -0.001	-	-
	HPLC	2	-0.20	-0.29 to -0.12	0.0	0.44
	ELISA	2	-0.14	-0.31 to 0.03	26.0	0. 24
HAZ	Overall	12	-0.17	-0.30 to -0.03	97.2	< 0.001
	Prospective cohort	8	-0.05	-0.10 to -0.01	58.2	0.01
	Non-cohort	4	-0.33	-0.75 to 0.10	99.0	< 0.001
	HPLC	6	-0.02	-0.10 to 0.05	73.3	0.002
	ELISA	4	-0.31	-0.70 to 0.08	98.9	< 0.001
	TLC	1	-0.46	-0.69 to -0.22	-	-
	IDMS	1	-0.18	-0.32 to -0.04	-	-
	Adjusted	11	-0.14	-0.29 to -0.001	97.4	< 0.001
	Not-adjusted	1	-0.46	-0.69 to -0.22	-	-
WAZ	Overall	8	-0.18	-0.33 to -0.02	97.9	< 0.001
	Prospective cohort	5	-0.05	-0.07 to -0.03	4.3	0.38
	Non-cohort	3	-0.32	-0.69 to 0.05	96.7	< 0.001
	HPLC	3	-0.06	-0.11 to -0.02	41.1	0.18
	ELISA	3	-0.33	-0.78 to 0.12	99.3	< 0.001
	TLC	1	-0.20	-0.48 to 0.08	-	-
	IDMS	1	-0.03	-0.17 to 0.11	-	-
	Adjusted	7	-0.17	-0.34 to -0.01	98.2	< 0.001
	Not-adjusted	1	-0.20	-0.48 to 0.08	43.2	0.17
WHZ	Overall	6	-0.02	-0.07 to 0.03	43.6	0.11
	Prospective cohort	4	-0.01	-0.07 to 0.04	59.6	0.06
	Non-cohort	2	-0.07	-0.27 to 0.13	18.7	0.26
	HPLC	4	-0.01	-0.05 to 0.03	0.0	0.50
	ELISA	1	-0.06	-0.10 to -0.02	-	-
	IDMS	1	0.09	-0.04 to 0.22	-	-
**Dichotomous outcome**	**Subgroup**	**Studies**	**Odds ratio**	**95%CI**	**I**^**2**^** (%)**	**P**
Stunting	Overall	6	1.15	0.98 to 1.35	80.3	< 0.001
	Prospective cohort	3	1.21	1.11 to 1.33	0.0	0.50
	Non-cohort	3	1.14	0.80 to 1.63	43.5	0.15
Underweight	Overall	3	1.08	0.92 to 1.27	70.6	0.03
	Prospective cohort	1	1.20	1.03 to 1.40	-	-
	Non-cohort	2	0.99	0.98 to 1.00	0.0	0.37
Wasting	Overall	1	1.00	0.98 to 1.02	-	-

*WHZ* Weight-for-height z score, *WAZ* Weight-for-age z-score, *HAZ* Height-for-age z-scores, *HPLC* High-performance liquid chromatography, *ELISA* Enzyme-linked immunosorbent assay, *TLC* Thin layer chromatography, *IDMS* Isotope dilution mass spectrometry

**Fig. 6:**
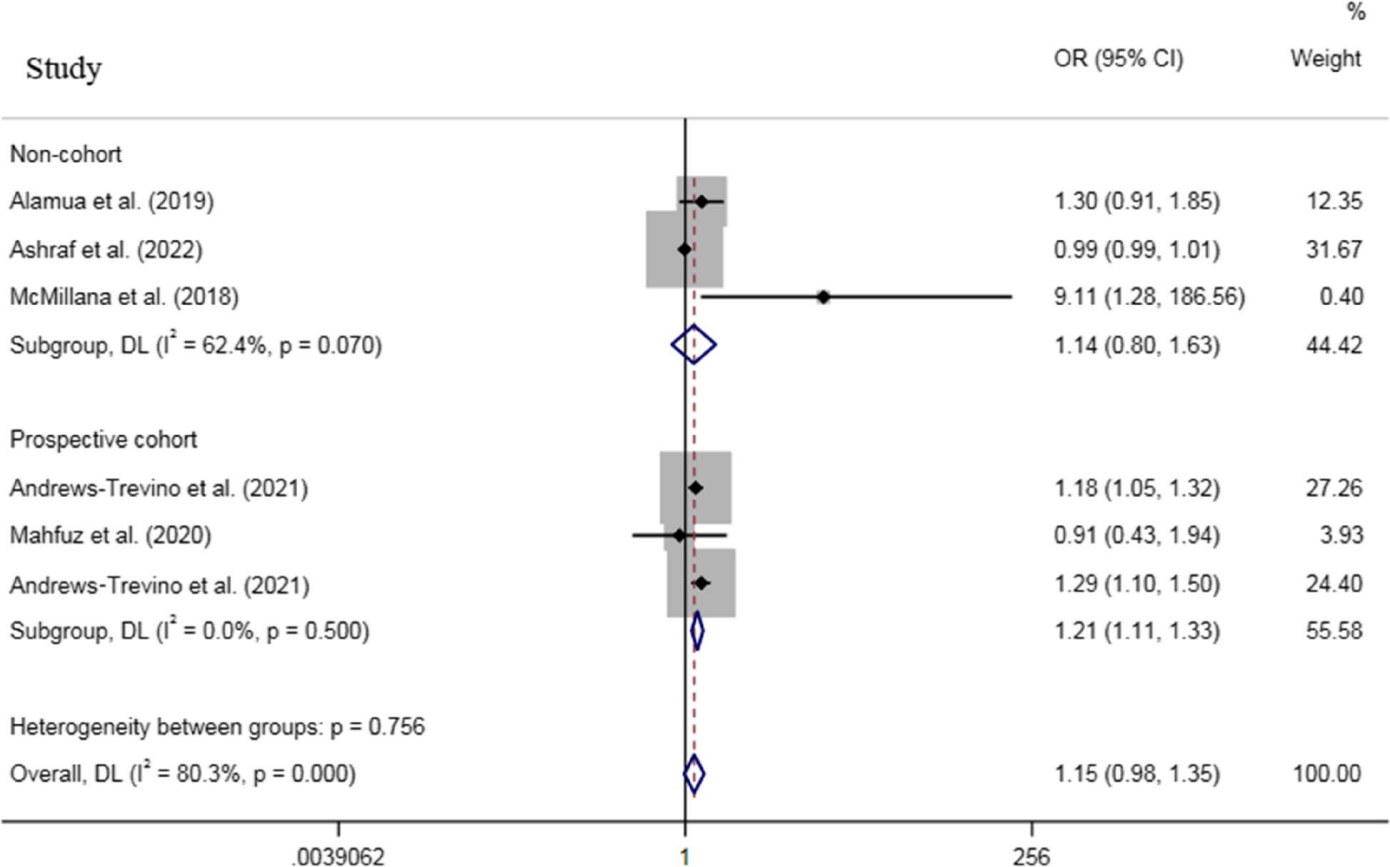
Meta‐analysis of the association between exposure to aflatoxin B1 and risk of stunting stratified by study design (odds ratio and 95% confidence interval)

**Fig. 7:**
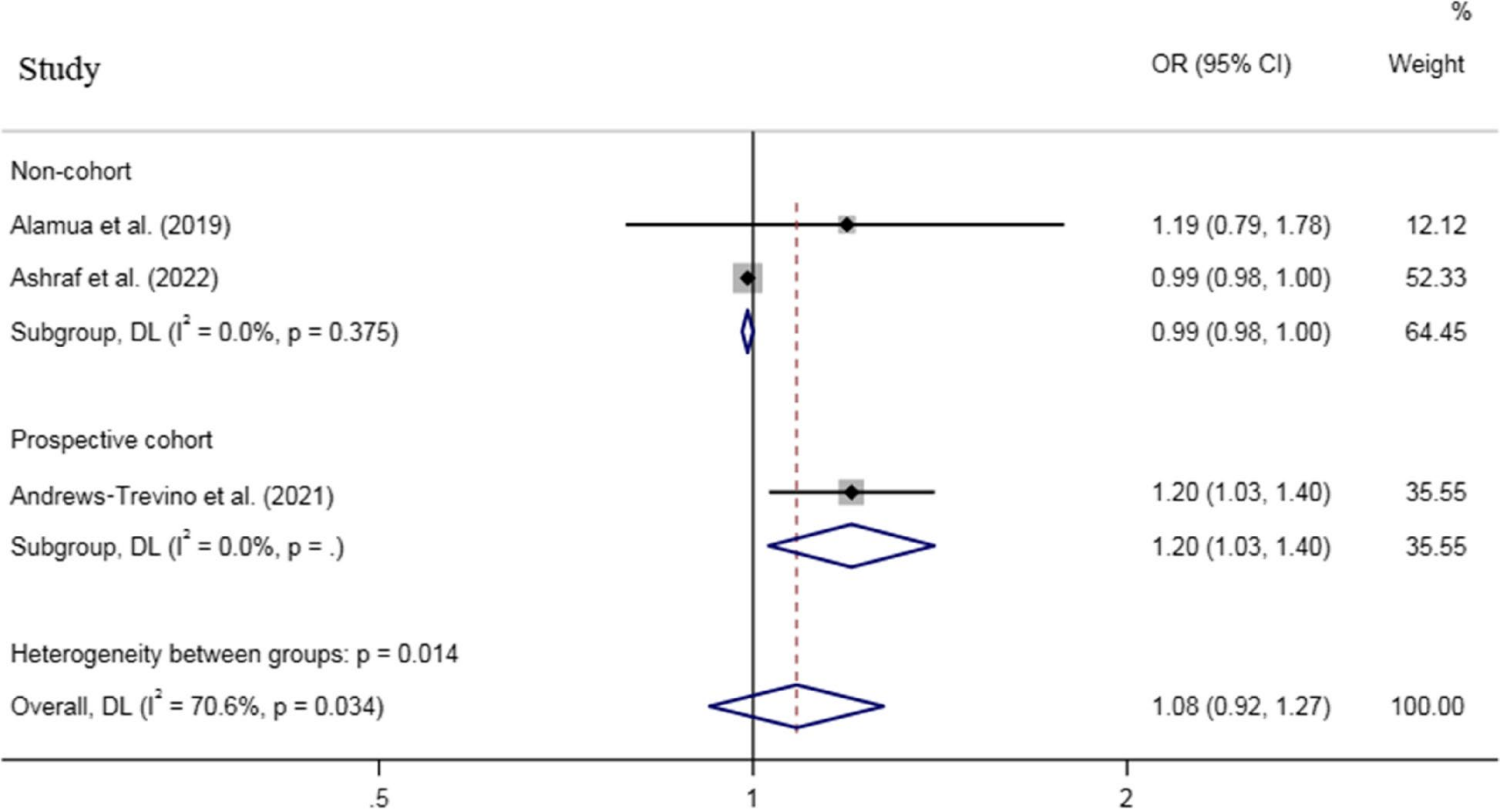
Meta‐analysis of the association between exposure to aflatoxin B1 and risk of underweight stratified by study design (odds ratio and 95% confidence interval)

For dichotomous outcomes, an increased odds was observed for stunting (Fig. 6) and underweight (Fig. 7), while no association was found between AFB1 exposure and wasting (based on 1 study) when all effect sizes were pooled, with a remarkable heterogeneity across studies (P_heterogeneity_ for stunting < 0.001, P_heterogeneity_ for underweight = 0.03) (Table 2).

## Discussion

The current study is the first systematic review and meta-analysis which aimed to investigate the association of aflatoxin B1 (AFB1) exposure and growth failure in children. In our study, based on 17 studies with a total sample size of 5,633 participants, pooled analyzes of all available effect sizes found an inverse association between exposure to AFB1 and growth failure measurements (height, HAZ, WAZ, and WHZ, underweight, and stunting) in children. Similarly to our results, Voth-Gaeddert et al., in 2018, confirmed a significant negative correlation between putative aflatoxin exposure levels and child HAZ [44].

The association of AFB1to growth of infants/children has been inconsistent. The cross-sectional study by Chen et al., in 2018, on 114 children under 36 months of age in Tanzania, indicated that, no associations were found between aflatoxin exposures and growth impairment as measured by stunting, underweight [WAZ <  − 2], or wasting [WHZ <  − 2] [20]. Moreover, in agreement with our study, a longitudinal study on 200 children 16–37 months of age indicated that there was a strong negative correlation between serum aflatoxin–albumin (AF-alb) and height increase over the 8-month follow-up after adjustment for age, sex, height at recruitment, socioeconomic status, and weaning status. Also, this study emphasizes the association between aflatoxin and stunting, although the underlying mechanisms remain unclear [45].

Uncertainty analysis by Rasheed et al., in 2021, revealed that reducing aflatoxin exposure in high exposure areas up to non-detectable levels could save the stunting disability-adjusted life years (DALYs) up to 50%. The burden of childhood all causes stunting is greater in countries with higher aflatoxin exposure such as Benin [46]. Kiarie et al., in 2016, reported that there was no association between total aflatoxins (aflatoxin B and aflatoxin G) and HAZ, WAZ and WHZ [47]. A cluster randomized controlled design study on 1230 unborn children in Eastern Kenya indicated that reducing AF exposure through a swapping and stockist intervention significantly lowered serum AF levels: at study endline (24 months after study enrolment during pregnancy), children in intervention communities had serum AF levels that were 27% lower than in the control communities [48]. The inconsistencies in the results of the available studies may be due to the differences in study design, duration of exposure, age of subjects, and the level of adjustment for covariates.

Aflatoxins could potentially impact the growth of children via three different mechanisms; it is believed that it may be involved in enteropathy. The damage caused by aflatoxin to the epithelium of the intestine could result in the loss of nutrients [49]. Additionally, there is a possibility that aflatoxin can suppress the immune system, increasing the susceptibility to infections, including diarrhea, which consequently reduces nutrient uptake [49] The immune suppression effect of aflatoxin has been observed in some animals and humans [50]. Lastly, chronic exposure to aflatoxin could lead to liver toxicity, causing damage to the insulin-like growth factor pathway proteins (IGFs), resulting in a decrease of IGFs in circulation which ultimately affects child growth in an unfavorable manner [12, 51]. A recent study on human liver cells identified that aflatoxin suppressed IGFs gene/protein expressions in a dose-dependent pattern [35]. The data supported that aflatoxin induced variations in IGFs could play a role in growth failure [52]. Overall, our study suggest that reducing exposure to aflatoxin is a potential strategy to decrease the burden of growth failure in children/infants. Some strategies to decrease exposure to aflatoxins and mitigate the risk of exposure include good agricultural practices (GAPs) to prevent fungal growth and contamination of crops [53], proper storage of crops to prevent moisture and fungal growth [54], simple food preparation methods such as sorting, washing, crushing, and dehulling [55], adopting chemical methods, such as treatment with organic acids, ammonia, ozone or enzymes to minimize the levels of aflatoxins present in food, and drying crops to reduce moisture content and prevent fungal growth [56]. In addition to these strategies, monitoring and preventive programs can decrease the risk of exposure in humans [53]. Sequestering aflatoxins in the gastrointestinal tract and reducing their bioavailability can also mitigate the risk of exposure [57]. Reporting any non-compliant food products to food safety authorities is also an important measure to prevent exposure to aflatoxins [55]. By implementing these strategies and continuing to research the impact of aflatoxin exposure on human health, we can work towards reducing the burden of this public health problem. It is important to note that the most effective intervention is prevention. Therefore, it is essential to implement GAPs and proper storage methods to prevent fungal growth and contamination of crops [55]. 

## Limitations

This is the first meta-analysis evaluating the relation of AFB1 exposure to growth indicators of children. The limitations of this meta-analysis should be declared. First, a significant evidence for heterogeneity was found across studies on HAZ and WAZ. Subgroup analysis identified the method used to assess AFB1, the level of adjustment for covariates, type of exposure (dietary vs. serum), and study design as sources of the observed heterogeneity. Second, some of the analyzed studies were cross-sectional in design, which are more susceptible to selection/recall biases compared to cohort publications. Third, because of the lack of adequate information in original studies, stratified analysis by sex was not carried out to evaluate likely gender-specific relationships. Another weakness of this study was the small number of the included studies, thus, publication bias analysis was not performed based on guidelines [58]. The results obtained for wasting was based on 1 study and should be interpreted with caution. Moreover, although the results of most studies were adjusted for potential confounding variables, the results of some studies were crude and unadjusted, which may lead to bias.

## Conclusions

Aflatoxin exposure is common in developing countries, making it an issue of substantial public health importance. Overall, this meta-analysis indicated that there existed a negatively association between AFB1 exposure and growth indicators of children. Therefore, it is essential develop preventive approaches, such as implementing GAPs and improving proper storage methods to prevent fungal growth and contamination of crops, and thus reduce the burdens of aflatoxins on health [55].

### Supplementary Information

**Additional file 1.**

**Additional file 2.**

**Additional file 3.**

**Additional file 4.**

## Data Availability

The study is based on extracting data from published articles and all data are included in the report.

## Abbreviations

AFB1: Aflatoxin B1
HAZ: Height-for-age z-score
WAZ: Weight-for-age z-score
WHZ: Weight-for-height z-score
OR: Odds ratios (OR)
95%CI: 95% Conference intervals
AFs: Aflatoxins
WHO: World Health Organization
IGF-1: Insulin-like growth factor 1
ELISA: Enzyme-linked immunosorbent assay
HPLC: High-performance liquid chromatography
TLC: Thin layer chromatography
AF-alb: Aflatoxin–albumin
DALYs: Disability-adjusted life years

## References

[1] Eskola M, Kos G, Elliott CT, Hajšlová J, Mayar S, Krska R (2020). Worldwide contamination of food-crops with mycotoxins: Validity of the widely cited ‘FAO estimate’ of 25%. Crit Rev Food Sci Nutr.

[2] Cardwell K (2000). Mycotoxin contamination of foods in Africa: Antinutritional factors. Food Nutr Bull.

[3] Park J, Kim E, Shon D, Kim Y (2002). Natural co-occurrence of aflatoxin B1, fumonisin B1 and ochratoxin A in barley and corn foods from Korea. Food Addit Contam.

[4] Turner PC, Moore SE, Hall AJ, Prentice AM, Wild CP (2003). Modification of immune function through exposure to dietary aflatoxin in Gambian children. Environ Health Perspect.

[5] Ahmed Adam MA, Tabana YM, Musa KB, Sandai DA (2017). Effects of different mycotoxins on humans, cell genome and their involvement in cancer (Review). Oncol Rep.

[6] Gong YY, Turner PC, Hall AJ, Wild CP (2008). Aflatoxin exposure and impaired child growth in West Africa: An unexplored international public health burden.

[7] Kinyoki DK, Osgood-Zimmerman AE, Pickering BV, Schaeffer LE, Marczak LB, Lazzar-Atwood A (2020). Mapping child growth failure across low- and middle-income countries. Nature.

[8] Victora CG, Adair L, Fall C, Hallal PC, Martorell R, Richter L (2008). Maternal and child undernutrition: consequences for adult health and human capital. The lancet.

[9] Xu Y, Gong Y, Routledge M (2018). Aflatoxin exposure assessed by aflatoxin albumin adduct biomarker in populations from six African countries. World Mycotoxin Journal.

[10] Lombard MJ. Mycotoxin Exposure and Infant and Young Child Growth in Africa: What Do We Know? Annals of Nutrition and Metabolism. 2014;64(suppl 2):42–52. 10.1159/000365126 25341872

[11] Wild CP, Miller JD, Groopman JD. Mycotoxin control in low-and middle-income countries. 2015. 27030861

[12] Smith LE, Stoltzfus RJ, Prendergast A (2012). Food chain mycotoxin exposure, gut health, and impaired growth: a conceptual framework. Adv Nutr.

[13] Smith JW, Matchado AJ, Wu LS, Arnold CD, Burke SM, Maleta KM, et al. Longitudinal Assessment of Prenatal, Perinatal, and Early-Life Aflatoxin B(1) Exposure in 828 Mother-Child Dyads from Bangladesh and Malawi. Current developments in nutrition. 2022;6(2):nzab153. 10.1093/cdn/nzab153 PMC8829025 35155983

[14] Watson S, Moore SE, Darboe MK, Chen G, Tu Y-K, Huang Y-T (2018). Impaired growth in rural Gambian infants exposed to aflatoxin: a prospective cohort study. BMC Public Health.

[15] Turner PC, Collinson AC, Cheung YB, Gong Y, Hall AJ, Prentice AM (2007). Aflatoxin exposure in utero causes growth faltering in Gambian infants. Int J Epidemiol.

[16] McMillan A, Renaud JB, Burgess KM, Orimadegun AE, Akinyinka OO, Allen SJ (2018). Aflatoxin exposure in Nigerian children with severe acute malnutrition. Food Chem Toxicol.

[17] Nabwire Wangia-Dixon R, Xue KS, Alcala J, Quach THT, Song X, Tang L (2020). Nutrition and growth outcomes are affected by aflatoxin exposures in Kenyan children. Food Addit Contam Part A Chem Anal Control Expo Risk Assess.

[18] Leroy JL, Sununtnasuk C, García-Guerra A, Wang JS (2018). Low level aflatoxin exposure associated with greater linear growth in southern Mexico: A longitudinal study. Matern Child Nutr.

[19] Daou R, Hoteit M, Bookari K, Al-Khalaf M, Nahle S, Al-Jawaldeh A (2022). Aflatoxin B1 Occurrence in Children under the Age of Five&rsquo;s Food Products and Aflatoxin M1 Exposure Assessment and Risk Characterization of Arab Infants through Consumption of Infant Powdered Formula: A Lebanese Experience. Toxins.

[20] Chen C, Mitchell NJ, Gratz J, Houpt ER, Gong Y, Egner PA (2018). Exposure to aflatoxin and fumonisin in children at risk for growth impairment in rural Tanzania. Environ Int.

[21] Tessema M, De Groote H, Brouwer ID, De Boevre M, Corominas AV, Stoecker BJ (2021). Exposure to aflatoxins and fumonisins and linear growth of children in rural Ethiopia: a longitudinal study. Public Health Nutr.

[22] Mitchell NJ, Hsu HH, Chandyo RK, Shrestha B, Bodhidatta L, Tu YK (2017). Aflatoxin exposure during the first 36 months of life was not associated with impaired growth in Nepalese children: An extension of the MAL-ED study. PLoS ONE.

[23] Hoffmann V, Jones K, Leroy J (2018). The impact of reducing dietary aflatoxin exposure on child linear growth: a cluster randomised controlled trial in Kenya. BMJ Glob Health.

[24] Moher D, Liberati A, Tetzlaff J, Altman DG (2009). Preferred reporting items for systematic reviews and meta-analyses: the PRISMA statement. PLoS Med.

[25] Peterson J, Welch V, Losos M, Tugwell PJ. The Newcastle-Ottawa scale (NOS) for assessing the quality of nonrandomised studies in meta-analyses. Ottawa: Ottawa Hospital Research Institute. 2011;2(1):1–2.

[26] Mozaffari H, Ajabshir S, Alizadeh S (2020). Dietary Approaches to Stop Hypertension and risk of chronic kidney disease: A systematic review and meta-analysis of observational studies. Clin Nutr.

[27] Shamseer L, Moher D, Clarke M, Ghersi D, Liberati A, Petticrew M, Preferred reporting items for systematic review and meta-analysis protocols (PRISMA-P),  (2015). elaboration and explanation. BMJ.

[28] Pourhassan B, Meysamie A, Alizadeh S, Habibian A, Beigzadeh Z (2019). Risk of obstructive pulmonary diseases and occupational exposure to pesticides: a systematic review and meta-analysis. Public Health.

[29] Mohseni R, Mohseni F, Alizadeh S, Abbasi S (2020). The association of dietary approaches to stop hypertension (DASH) diet with the risk of colorectal cancer: a meta-analysis of observational studies. Nutr Cancer.

[30] Emami MR, Safabakhsh M, Alizadeh S, Asbaghi O, Khosroshahi MZ (2019). Effect of vitamin E supplementation on blood pressure: a systematic review and meta-analysis. J Hum Hypertens.

[31] Alizadeh S, Esmaeili H, Alizadeh M, Daneshzad E, Sharifi L, Radfar H (2019). Metabolic phenotypes of obese, overweight, and normal weight individuals and risk of chronic kidney disease: a systematic review and meta-analysis. Archives of endocrinology and metabolism.

[32] DerSimonian R, Laird N (1986). Meta-analysis in clinical trials. Control Clin Trials.

[33] Gong Y, Egal S, Hounsa A, Turner P, Hall A, Cardwell K (2003). Determinants of aflatoxin exposure in young children from Benin and Togo, West Africa: the critical role of weaning. Int J Epidemiol.

[34] Shouman BO, El Morsi D, Shabaan S, Abdel-Hamid A-H, Mehrim A (2012). Aflatoxin B1 level in relation to child’s feeding and growth. The Indian Journal of Pediatrics.

[35] Castelino JM, Routledge MN, Wilson S, Dunne DW, Mwatha JK, Gachuhi K (2015). Aflatoxin exposure is inversely associated with IGF1 and IGFBP3 levels in vitro and in Kenyan schoolchildren. Mol Nutr Food Res.

[36] Shirima CP, Kimanya ME, Routledge MN, Srey C, Kinabo JL, Humpf H-U (2015). A prospective study of growth and biomarkers of exposure to aflatoxin and fumonisin during early childhood in Tanzania. Environ Health Perspect.

[37] Alamu EO, Gondwe T, Akello J, Maziya-Dixon B, Mukanga M (2020). Relationship between serum aflatoxin concentrations and the nutritional status of children aged 6–24 months from Zambia. Int J Food Sci Nutr.

[38] Andrews-Trevino JY, Webb P, Shively G, Kablan A, Baral K, Davis D (2021). Aflatoxin exposure and child nutrition: measuring anthropometric and long-bone growth over time in Nepal. Am J Clin Nutr.

[39] Mahfuz M, Hasan ST, Alam MA, Das S, Fahim SM, Islam MM (2021). Aflatoxin exposure was not associated with childhood stunting: results from a birth cohort study in a resource-poor setting of Dhaka. Bangladesh Public health nutrition.

[40] Andrews-Trevino J, Webb P, Shrestha R, Pokharel A, Acharya S, Chandyo R (2022). Exposure to multiple mycotoxins, environmental enteric dysfunction and child growth: Results from the AflaCohort Study in Banke. Nepal Maternal & Child Nutrition.

[41] Ashraf W, Rehman A, Ahmad M-U-D, Rabbani M, Mushtaq MH, Aamir K, et al. Assessment of aflatoxin B1-lysine adduct in children and its effect on child growth in Lahore, Pakistan. Food Additives & Contaminants: Part A. 2022;39(8):1463–73. 10.1080/19440049.2022.2080871 35652855

[42] Matchado A, Smith JW, Schulze KJ, Groopman JD, Kortekangas E, Chaima D, et al. Child aflatoxin exposure is associated with poor child growth outcomes: a prospective cohort study in rural Malawi. Curr Develop Nutri. 2023:101962. 10.1016/j.cdnut.2023.101962 PMC10328803 37426291

[43] Haddaway NR, Page MJ, Pritchard CC, McGuinness LA (2022). PRISMA2020: An R package and Shiny app for producing PRISMA 2020-compliant flow diagrams, with interactivity for optimised digital transparency and Open Synthesis. Campbell Syst Rev.

[44] Voth-Gaeddert LE, Stoker M, Torres O, Oerther DB (2018). Association of aflatoxin exposure and height-for-age among young children in Guatemala. Int J Environ Health Res.

[45] Gong Y, Hounsa A, Egal S, Turner PC, Sutcliffe AE, Hall AJ (2004). Postweaning exposure to aflatoxin results in impaired child growth: a longitudinal study in Benin. West Africa Environmental health perspectives.

[46] Rasheed H, Xu Y, Kimanya ME, Pan X, Li Z, Zou X (2021). Estimating the health burden of aflatoxin attributable stunting among children in low income countries of Africa. Sci Rep.

[47] Kiarie G, Dominguez-Salas P, Kang’ethe S, Grace D, Lindahl J. Aflatoxin exposure among young children in urban low-income areas of Nairobi and association with child growth. Afr J Food Agricult Nutri Develop. 2016;16(3):10967–90.

[48] Hoffmann V, Jones K, Leroy JL (2018). The impact of reducing dietary aflatoxin exposure on child linear growth: a cluster randomised controlled trial in Kenya. BMJ Glob Health.

[49] Watson S, Gong YY, Routledge M (2017). Interventions targeting child undernutrition in developing countries may be undermined by dietary exposure to aflatoxin. Crit Rev Food Sci Nutr.

[50] Lizárraga-Paulín EG, Moreno-Martínez E, Miranda-Castro SP (2011). Aflatoxins and their impact on human and animal health: an emerging problem. Aflatoxins-Biochemistry and molecular biology.

[51] Gong YunYun GY, Turner PC, Hall AJ, Wild CP. Aflatoxin exposure and impaired child growth in West Africa: an unexplored international public health burden? Mycotoxins: detection methods, management, public health and agricultural trade: Cabi Wallingford UK; 2008. p. 53–65.

[52] Gong YY, Watson S, Routledge MN (2016). Aflatoxin exposure and associated human health effects, a review of epidemiological studies. Food safety.

[53] Negash D (2018). A review of aflatoxin: occurrence, prevention, and gaps in both food and feed safety. Journal of Applied Microbiological Research.

[54] Jallow A, Xie H, Tang X, Qi Z, Li P (2021). Worldwide aflatoxin contamination of agricultural products and foods: From occurrence to control. Comprehensive reviews in food science and food safety.

[55] Strosnider H, Azziz-Baumgartner E, Banziger M, Bhat RV, Breiman R, Brune M-N (2006). Workgroup report: public health strategies for reducing aflatoxin exposure in developing countries. Environ Health Perspect.

[56] Wild CP, Miller JD, Groopman JD. Intervention strategies to reduce human exposure to aflatoxins and fumonisins. Mycotoxin control in low-and middle-income countries. 2015.

[57] Magnussen A, Parsi MA (2013). Aflatoxins, hepatocellular carcinoma and public health. World J Gastroenterol: WJG.

[58] Tarsilla M (2010). Cochrane handbook for systematic reviews of interventions. Journal of Multidisciplinary Evaluation.

