# Supplementary material for: A systematic review with meta-analysis of the relation of aflatoxin B1 to growth impairment in infants/children
Source: BMC Pediatr. 2023 Dec 5;23:614. doi: 10.1186/s12887-023-04275-9 (PMC10696779; doi:10.1186/s12887-023-04275-9)
Supplement: Supplementary file 2 — Additional file 2. [file 12887_2023_4275_MOESM2_ESM.docx]

Search strategy:

((((((((((("Aflatoxins"[Majr]) OR ("Mycotoxins"[Mesh])) OR (Aflatoxins[Title/Abstract])) OR (Aflatoxin[Title/Abstract])) OR (Mycotoxins[Title/Abstract])) OR (Mycotoxin[Title/Abstract])) OR (aflatoxin B1[Title/Abstract])) OR (AFB1[Title/Abstract])) OR (aflatoxin B1-lysine[Title/Abstract])) OR (AFB1-lysine[Title/Abstract])) OR (AFB1-lys[Title/Abstract])) OR (AFB1-lys[Title/Abstract])) AND ((((((("Child"[Mesh]) OR ("Infant"[Mesh])) OR (child[Title/Abstract])) OR (Infant[Title/Abstract])) OR (children[Title/Abstract])) OR (infants[Title/Abstract])) OR (neonatal[Title/Abstract]))) AND ((((((((((((((((growth[Title/Abstract]) OR (weight[Title/Abstract])) OR (length[Title/Abstract])) OR (height[Title/Abstract])) OR (anthropometry[Title/Abstract])) OR (length-for-age[Title/Abstract])) OR (height-for-age[Title/Abstract])) OR (weight-for-age[Title/Abstract])) OR (weight-for-length[Title/Abstract])) OR (weight-for-height[Title/Abstract])) OR (stunting[Title/Abstract])) OR (wasting[Title/Abstract])) OR (underweight[Title/Abstract])) OR ("Growth"[Mesh])) OR (Body Height"[Majr])) OR ("Child Development"[Mesh])).
