# Supplementary material for: A systematic review with meta-analysis of the relation of aflatoxin B1 to growth impairment in infants/children
Source: BMC Pediatr. 2023 Dec 5;23:614. doi: 10.1186/s12887-023-04275-9 (PMC10696779; doi:10.1186/s12887-023-04275-9)
Supplement: Supplementary file 3 — Additional file 3. [file 12887_2023_4275_MOESM3_ESM.docx]

Supplementary file 2

Mathematical calculation methods for standardized regression coefficient (β):

Nieminen et al (1) presented how to calculate the standardized regression coefficient β and its standard error. The calculation methods are briefly described as follows.

1. Standardized regression coefficient and standard error were extracted directly if reported in the original studies. Then the standard error was measured based on the confidence interval.
2. If Pearson or Spearman correlation analysis was done in a study, the correlation coefficient r was considered as β and its standard error was calculated based on the following relationship.


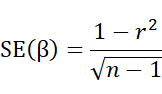


Equation S.1

1. If only a simple linear regression or a multivariate linear regression model was performed in the study, the adjusted regression coefficient was converted to the standardized regression coefficient and the corresponding standard error using the following formulas:


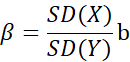


Equation S.2


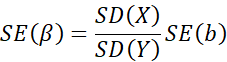


Equation S.3

where SD(X), SD(Y), “b” and SE(b) demonstrated the standard deviation of exposure variable, the standard deviation of response variable, the adjusted regression coefficient or simple regression coefficient, and standard error for b.

1. In studies that compared the response rate between exposure groups (low and high exposures), the following formulas were used to determine the standard regression coefficient and its standard error.


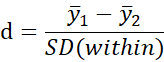


Equation S.4


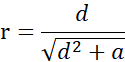


Equation S.5


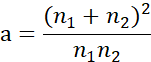


Equation S.6


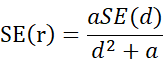


Equation S.7

d= mean difference effect size

y_1_, y_2=_ mean response values in the low and high exposure groups

SD= within-group standard deviation

a = correction factor

1. Nieminen P. Application of Standardized Regression Coefficient in Meta-Analysis. BioMedInformatics. 2022;2(3):434-58.
