# Supplementary material for: A systematic review with meta-analysis of the relation of aflatoxin B1 to growth impairment in infants/children
Source: BMC Pediatr. 2023 Dec 5;23:614. doi: 10.1186/s12887-023-04275-9 (PMC10696779; doi:10.1186/s12887-023-04275-9)
Supplement: Supplementary file 4 — Additional file 4. [file 12887_2023_4275_MOESM4_ESM.docx]

**Online Supporting Material**

**Supplemental Table 1.** Quality assessment of studies included in this systematic review and meta-analysis^1^

| **Prospective studies** | Representativeness of the exposed cohort | Selection of the non-exposed cohort | Ascertainment of exposure | Demonstration that outcome of interest was not present at start of study | Study controls for age | Study controls for any additional factor | Assessment of outcome | Was follow-up long enough for outcomes to occur ≥1 year) | Adequacy of follow up of cohorts (loss-to-follow up <20%) | **Total score** |
| --- | --- | --- | --- | --- | --- | --- | --- | --- | --- | --- |
| Andrews-Trevino et al., 2021 | * | * | * | * | * | * | * | * | * | 9 |
| Mahfuz et al., 2020 | * | * | * | * | NA | * | * | * | * | 8 |
| Andrews-Trevino et al., 2021 | * | * | * | * | NA | * | * | NA | * | 7 |
| Shirima et al., 2015 | * | * | * | * | * | * | * | * | * | 9 |
| Tessema et al., 2021 | * | * | * | NA | * | * | * | * | NA | 7 |
| Watson et al., 2018 | * | * | * | NA | * | * | * | * | * | 8 |
| Turner et al., 2007 | * | * | * | NA | * | * | * | * | * | 8 |
| Matchado et al., 2023 | * | * | * | NA | * | * | * | * | * | 8 |
| **Cross-sectional studies** | Representativeness of the sample | Sample size | Non-respondents | Ascertainment of exposure | Study controls for age | Study controls for any additional factor | Ascertainment of outcome | Statistical test |  | **Total score** |
| Alamua et al. 2019 | * | * | NA | * | * | * | ** | * |  | 8 |
| Ashraf et al., 2022 | * | * | NA | * | NA | * | ** | * |  | 7 |
| Gong et al., 2003 | * | * | NA | * | * | * | ** | * |  | 8 |
| McMillana et al., 2018 | * | NA | NA | * | * | * | ** | * |  | 7 |
| Wangia-Dixon et al., 2020 | * | * | NA | * | * | * | ** | * |  | 8 |
| Shouman et al., 2011 | * | NA | NA | * | NA | NA | ** | * |  | 4 |
| Castelino et al., 2015 | * | * | NA | * | * | * | ** | * |  | 8 |

**^1^**According to the Newcastle-Ottawa Scale (NOS) criteria

NA: Not Applicable
